# Outcome Assessment of a Dedicated HIV Positive Health Care Worker Clinic at a Central Hospital in Malawi: A Retrospective Observational Study

**DOI:** 10.1371/journal.pone.0019789

**Published:** 2011-05-19

**Authors:** Adrienne K. Chan, Monique van Lettow, Lyson Tenthani, Moses Kumwenda, Lucy Gawa, Alice Kadzanja, Austin Mnthambala, Marion Kambanji

**Affiliations:** 1 Dignitas International, Zomba, Malawi; 2 Department of Medicine, St. Michael's Hospital, University of Toronto, Toronto, Canada; 3 Department of HIV and AIDS, Malawi Ministry of Health, Lilongwe, Malawi; 4 Dalla Lana School of Public Health, University of Toronto, Toronto, Canada; University of Cape Town, South Africa

## Abstract

**Background:**

Malawi has one of the world's lowest densities of Health Care Workers (HCW) per capita. This study evaluates outcomes of a dedicated HCW HIV clinic in Malawi, created at Zomba Central Hospital in January 2007.

**Methods and Findings:**

Retrospective cohort data was analyzed comparing HCW clinic patient baseline characteristics and treatment outcomes at 18 months after inception, against those attending the general HIV clinic. In-depth interviews and focus group discussions were conducted to explore perceptions of patients and caregivers regarding program value, level of awareness and barriers for uptake amongst HCW. 306 patients were enrolled on antiretroviral therapy (ART) in the HCW HIV clinic, 6784 in the general clinic. Significantly (p<0.01) more HCW clients were initiated on ART on the basis of CD4 as opposed to WHO Stage 3/4 (36% vs.23%). Significantly fewer HCW clients defaulted (6% vs.17%), and died (4% vs.12%). The dedicated HCW HIV clinic was perceived as important and convenient in terms of reduced waiting times, and prompt and high quality care. Improved confidentiality was an appreciated quality of the HCW clinic however barriers included fear of being recognized.

**Conclusions/Significance:**

Outcomes at the HCW clinic appear better compared to the general HIV clinic. The strategy of dedicated clinics to care for health providers is a means of HIV impact mitigation within human resource constrained health systems in high prevalence settings.

## Introduction

In sub-Saharan Africa, the profound lack of human resources for health (HRH) is a major barrier to the scale-up of universal antiretroviral therapy (ART) coverage [1], [2]. In Malawi, both the “brain drain” of professional health care workers (HCW) to foreign countries and the considerable number of staff who have complications of HIV-related disease are major contributors to the HRH crisis [3]. A 2007 national HRH census reported that there were only 159 doctors (1/100,000 pop.) and 3,614 nurses/midwifes/nurse-technicians (26/100,000 pop.) in the country, among the lowest in the region [4], [5]. In 2005, the annual attrition rate amongst Ministry of Health (MOH) staff stood at 6%, with 44% of losses caused by sickness and death, thought to be mainly due to HIV [6]. A 2007 survey in Malawi demonstrated that where HIV services have been accessed by HCW through public sector scale-up of ART, the outcomes have been good, with an estimated 250 HCW lives saved nationally, 12 months after ART initiation [7]. While there is some grey literature and case reports describing HCW initiatives in high HIV prevalence settings [8], [9], [10], [11], [12], very little has been published looking at outcomes of targeted clinics or programs that aim to increase HCW access to HIV related services.

One consequence of the success of ART scale up has been to further compound the workload issues faced by the health workforce [4]. In Zomba District (pop. 670,000) in Southern Malawi (adult HIV prevalence 16.5% [13]), MOH scale up of HIV related services began in 2004 and since inception has been supported by Dignitas International (DI), a Canadian humanitarian NGO. Initial scale-up began with the establishment of a HIV clinic at Zomba Central Hospital (ZCH), a tertiary level referral center, in October 2004. At ZCH there currently is a 94% physician vacancy rate and a 64% nursing vacancy rate [14]. Despite these HRH constraints, by the time this study was conducted, over 10,000 patients had been enrolled on ART through this partnership, with ART provision decentralized for district-wide geographical coverage to 20 rural health centers [15]. In 2006, the management at ZCH identified an urgent need to develop a targeted HIV care and treatment program for HCW that would remove barriers to ART access, provide confidentiality in testing and support, and that was conveniently located.

The purpose of this study was to evaluate the impact of this dedicated HCW HIV clinic and to draw lessons for further program development and inform policy. Specific objectives included measurement of the demographics and program outcomes of patients followed at the HCW HIV clinic and comparing them with individuals attending the general HIV clinic; assessment of the level of general awareness of the clinic amongst HCW who are the target of the intervention; and assessment of the perceptions of patients (current and potential), hospital management and care providers about the value and barriers to uptake of clinic services.

## Methods

### Study Setting and Description of Program Intervention

In late 2006, a multidisciplinary task force at ZCH involving the Hospital Director, the Chief Nursing Officer, the Deputy Director of Clinical Services and the DI HIV Clinic Coordinator created a program strategy for the dedicated HCW HIV clinic. The intervention, an HIV clinic dedicated to HCW and their immediate families, was one of the first of its kind in Malawi, with clinic inception in January 2007. This clinic was strategically placed physically separate from the general HIV clinic and was meant to be family-centered and provide integrated services including HIV specific services as well as non-HIV related primary care for both acute and chronic conditions for HCW living with HIV/AIDS.

The HCW HIV clinic space was located in a room in the low traffic, administrative area of the hospital, separated from regular outpatient clinic areas and inpatient wards. The HCW HIV Clinic was staffed by one dedicated nurse with specialty training and experience in HTC, PMTCT and ART Provision. The dedicated nurse is herself HIV seropositive and open about her status, and had asked specifically to be involved in providing peer support and counseling to her colleagues. The HIV Clinic Coordinator was designated to provide consultant services for the HCW HIV clinic.

Prior to clinic inception, sensitization activities regarding this service were conducted at ZCH and the Zomba District Health Office (DHO), as well as at the general HIV clinic. During general sensitization meetings and within hospital management circulars, the location of the clinic was not disclosed, however a mobile phone number was provided, and key contact people (ZCH and Zomba DHO Management, the DI Clinic Coordinator, the HCW clinic nurse and the general HIV clinic staff) were identified through whom referrals could be made.

The Task Force decided that the clinic would be open for all HCW in the district (including ZCH, the DHO, NGOs). Once a HCW had been tested and found to be seropositive, an invitation was extended by care providers to the staff to provide testing, treatment and care at the HCW HIV clinic to the staff, their partner and their children. Extended family was excluded from the HCW HIV clinic in order to maintain both confidentiality and to maintain the goal of having scheduled appointments and low waiting times. Families were encouraged to harmonize appointment times and been seen as a unit. Initially the clinic was operational on weekdays for 2 hours in the morning, but has since grown to operate for 5 hours for 5 days a week.

Services provided include: HTC, PMTCT, cotrimoxazole prophylaxis, post-exposure prophylaxis (PEP), staging and initiation of ART, management of HIV related illnesses and drug toxicities, and general integrated primary care for pre-ART and ART patients (i.e. diabetes, HTN, outpatient management of acute illness like malaria or diarrhea). Essential drugs and HIV related drugs are kept in a locked cabinet in the clinic area, so that medications can be dispensed to staff directly. The nurse performs phlebotomy for HIV related laboratory testing (CD4) and ensures that samples are delivered to the ZCH lab and results returned to the patients confidentially. All health records and program data are kept in a confidential program record separate from the general HIV clinic records. ART provision at the HCW HIV clinic is the same as at the General clinic, as per the Malawian MOH guidelines [16].

### Study Design and Data Collection

A retrospective cross sectional descriptive analysis was performed. Baseline patient characteristics were compared between patients who were initiated at the HCW HIV clinic and patients who were initiated in the general clinic. Treatment outcomes were assessed comparing patients followed at the HCW and general clinic at the time of censoring (December 31, 2008).

Program data was collected using MOH standardized registers and master cards from October 1, 2004 until December 31, 2008. Primary treatment outcomes are defined as: alive and on ART; died – for any reason while on ART; defaulted – a patient on ART who has not been seen at the ART provision facility for >3 months after intended date of follow up; stopped – a patient known to have stopped treatment for any reason during ART; transferred out – to another ART facility (i.e. not the general or HCW HIV clinic). Secondary treatment outcomes are defined as: side effect – any clinical diagnosis related to first line treatment (stavudine-lamivudine-nevirapine in fixed dose combination) adverse drug events (including peripheral neuropathy, hepatitis, rash, lipodystrophy, lactic acidosis, intolerance/hypersensitivity); substitution – patients who are placed on alternative first line regimens (containing single or dual substitution for zidovudine, tenofovir or efavirenz) due to treatment related drug toxicities; switching – is defined as patients who were placed on second line regimen (adults: zidovidine-lamivuidine, tenofovir and boosted lopinavir; children: didanosine, abacavir and boosted lopinavir) due to first line treatment failure.

Qualitative data was generated through Indepth Interviews (IDIs) and Focus Group Discussions (FGDs). Participants for the IDIs were purposefully selected by including all leading members of the district and hospital management (District Health Officer, Hospital Director, Deputy Director of Clinical Services, Human Resources Manager, Chief Nursing Officer), all HCW HIV clinic care providers (medical coordinator, 2 nurses) and a number of patients who attended and did not attend the HCW HIV clinic. Interviews continued until saturation of information was reached at 21 IDIs. Participants included in the FGDs were indentified through chain referral sampling and consisted of members of staff who did and did not attend the HCW HIV clinic. 3 FGDs were conducted; 1 FGD with male clinical staff (8 participants); 1 FGD with female clinical staff (7 participants) and 1 FDG with support staff (8 participants; 5 male, 3 female).

All qualitative data was translated and transcribed into MS Word documents verbatim.

This study received ethical approval by the Malawi National Health Sciences Research Committee. Written informed consent was obtained from all participants involved in the study.

### Analysis

Comparisons between groups were made using chi-square tests for categorical data and t-tests for continuous data with normal distribution (age and time on treatment). A value of p<0.05 was considered statistically significant. Multivariate logistic regression models were fitted with reported side effects, default and death as outcome variables, and being followed at the HCW HIV clinic as the main predictor. Adjustment was made for sex, age, reason for ART initiation and time on treatment, as these covariates were assumed to be confounding or interacting factors.

Analyses were conducted using SPSS 17.0 (SPSS, Inc., Chicago, IL, USA). Qualitative data transcripts were entered and coded using the NVIVO 7.0 (QSR International Inc., Southport, UK). After all the transcripts were coded, coding reports were produced, and each code was examined in detail for themes and patterns across the IDIs and FGDs.

## Results

### Demographic Characteristic of the HCW HIV Clinic Patients

**Figure 1:**
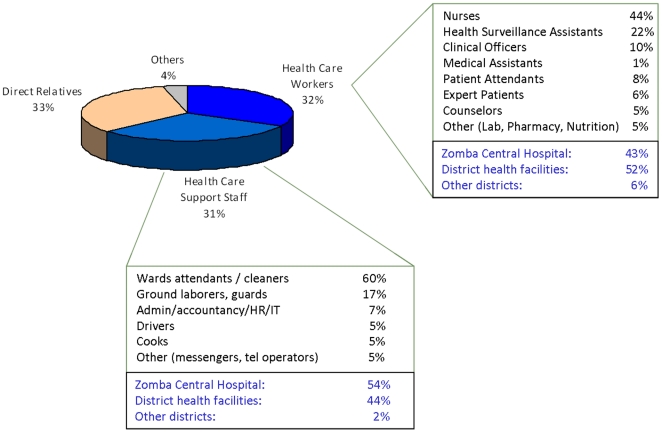
Occupational characteristics of ART patients in HCW clinic.

There were 7090 patients who ever started ART at ZCH by the time of censoring. Between Jan 1^st^, 2007 and December 31^st^, 2008, a total of 306 patients had been initiated on ART at the HCW HIV clinic and 6784 at the general HIV clinic. When reviewing the occupation of all ART patients initiated at the HCW HIV clinic, only 150/306 of the patients were recorded as HCW. **Figure 1** shows that out of all HCW client attendees, 63% could be defined as members of staff of the health system, 33% were related to health care staff members and 4% were neither member of staff nor related. 51% of staff members were HCW with direct patient care responsibilities (e.g. nurses, clinicians, pharmacists, lab technicians, lay health workers), while 49% of staff members were identified as health service support staff (e.g. ward attendants, cleaning staff, security guards, cooks, administrators). Of members of staff of the health system followed at the clinic, 48% worked at the district-level hospital where the clinic was located, 48% were stationed at peri-urban and rural health facilities within Zomba district, and 4% were working at health facilities in other districts.

**Table 1** demonstrates the baseline characteristics of patients ever initiated on ART in either the HCW HIV clinic or the general clinic. The mean age for patients initiating ART at the HCW HIV clinic was 37 years (± SD 12), compared to 33 years (± SD 14) at the general clinic; *p* = 0.01. A higher proportion of patients initiated because of low CD4 (and WHO Clinical Stage 1 or 2) as opposed to WHO Clinical Stage (3 or 4) at the HCW HIV clinic.

**Table 1:** Demographic characteristic of ART patients initiated in HCW clinic vs. general clinic.

Demographic Characteristics n(%)		HCW ClinicN = 306	ZCH (General) ClinicN = 6784	*p*-value
Sex	Female	61%	60%	0.58
Age Category	Under 15	24 (8%)	711 (10%)	0.03
	15 to 24	20 (7%)	729 (11%)	
	25 and over	262 (86%)	5344 (79%)	
ART Reason	Stage I Low CD4	1 (0.3%)	9 (0.1%)	<0.01
	Stage II Low CD4	109 (36%)	1570 (23%)	
	Stage III	140 (46%)	3739 (55%)	
	Stage IV	56 (18%)	1466 (22%)	

### Comparison of Treatment Outcomes of ART Patients in HCW HIV Clinic vs. General Clinic

At the time of analysis, of all patients on ART initiated in the HCW HIV clinic, 11(4%) had died, 1(0.3%) stopped, 8(3%) transferred out, 2(1%) defaulted, 15(5%) had missed their last appointment (potentially defaulted) and 9(3%) patients were decentralized to peripheral sites closer to their homes. Of all patient on ART initiated at the general clinic, 488(9%) had died, 20(0.3%) stopped, 684(10%) transferred out, 365(5%) defaulted, 614(9%) had missed their last appointment (potentially defaulted) and 1980(29%) patients were decentralized to peripheral sites closer to their homes. At time of censoring, the total number of patients alive and on treatment in the HCW HIV clinic and general clinic were 258 and 2533, respectively.

**Table 2** shows the treatment outcomes of patients followed on ART at the time of censoring in either the HCW HIV clinic or the general clinic. There was a significantly lower proportion of defaulting (6% vs. 17%; p<0.01) and death (4% vs. 12%; p<0.01) and a higher proportion of patients retained (87% vs. 58%; p<0.01) in the HCW HIV clinic. Significantly more side effects were reported in the HCW HIV Clinic compared to the general clinic. One in three patients in the HCW HIV clinic reported side effects, compared to one in seven in the general clinic. Significantly more HCW HIV clinic patients were changed from first line ART regimen to alternative first line regimens due to drug toxicity (22% vs. 7%; p<0.01) and second line regimen (2% vs. 0.3%) due to detected treatment failure. No significant difference was observed in reported side effects or type of regimen between HCW HIV clinic patients by level of cadre or whether they were involved directly in patient care vs. non-clinical support staff.

**Table 2:** Treatment outcomes in patients followed at HCW clinic vs. general clinic at time of study.

ART treatment Outcome		Health Care Worker ClinicN = 295	Zomba Central (General) ClinicN = 4345	*p*-value
Time on treatment	Mean number of months (±SD)	17.7 (±13.7)	10.4 (±10.6)	<0.01
Side effects	Reported Side effects	90 (31%)	643 (15%)	<0.01
Treatment Type	1^St^ Line	222 (75%)	3989 (92%)	<0.01
	1^st^ Line Alternative	65 (22%)	315 (7%)	
	2^nd^ Line	6 (2%)	13 (0.3%)	
	On Hold	1 (0.3%)	0.6 (.06%)	
Treatment Outcome	Defaulting	17 (6%)	759 (17%)	<0.01
	Died	11 (4%)	511 (12%)	
	Alive in Cohort	258 (87%)	2533 (58%)	
	Stopped	1 (0.3%)	19 (0.3%)	
	Transferred out	8 (3%)	523 (12%)	

**Table 3** shows multivariate analysis of factors associated with reported side effects, defaulting and death. Patients being followed at the HCW HIV clinic were more likely to report side effects [OR 1.85 (95% C.I. 1.40–2.44)] and less likely to be lost to follow-up (defaulted from treatment) [OR 0.14 (95% C.I. 0.03–0.56)], when adjusted for sex, age, clinical stage at ART initiation and time on ART. In multivariate analysis there was no association between being followed at the HCW HIV clinic and mortality [OR 0.61(95% C.I.0.32–1.16)].

**Table 3:** Factors associated with the occurrence of side effects, defaulting and death among patients being followed at the Health Care Worker clinic and general clinic in Zomba Central Hospital.

	SIDE EFFECTS		DEFAULTING		DEATH	
	Multivariate O.R. (95% C.I.)*	P-value	Multivariate O.R. (95% C.I.)*	P-value	Multivariate O.R. (95% C.I.)*	P-value
Followed at the HCW Clinic	1.85 (1.40–2.44)	0.001	0.14 (0.03–0.56)	0.005	0.61 (0.32–1.16)	0.13
Female **sex**	1.44 (1.21–1.71)	0.001	1.33 (1.05–1.69)	0.001	0.86 (0.71–1.04)	0.12
**Age** (per year)	1.04 (1.03–1.05)	0.01	1.02 (1.01–1.03)	0.001	1.01 (1.00–1.02)	0.009
**ART Reason**						
Stage I/II Low CD4	0.92 (0.73–1.16)	0.46	0.16 (0.10–0.24)	0.001	0.14 (0.10–0.19)	0.001
Stage III	0.74 (0.61–0.90)	0.003	0.57 (0.47–0.73)	0.001	0.48(0.39–0.59)	0.001
Stage IV	1.00		1.00		1.00	
**Time on treatment** (per month)	1.03 (1.02–1.04)	0.001	0.95 (0.93–0.96)	0.009	0.88 (0.87–0.90)	0.001

*adjusted for all other variables in the model.

### Level of Awareness of Potential Beneficiaries of the HCW HIV Clinic

Respondents, including those not attending, were aware of the HCW HIV clinic, services and location. Participants reported to have known about the clinic for up to two years and were able to describe the available services. Informal discussions through word of mouth from other HIV positive HCW were the most common means of sensitization about clinic services, with a second important means through referral from the general clinic or HTC services at other sites. Few participants mentioned the general sensitizations or management circular letters.

### Perceived Value of HCW HIV Clinic

Respondents reported that the HCW HIV clinic was important and convenient in terms of reduced waiting times and prompt and high quality confidential care: “… *during the time that we were using the general clinic, it was difficult …to go…get medicine and go back to work…the staff clinic is better because you are given appointments at specific times…when you go there… you can go back to your job in good time to continue your work*” (Attending Client IDI); “*…it is not easy to go to the clinic where every patient goes because … you are working at the same institution and those patients are the patients you serve…to be on the queue with them is not easy…But if you have your own clinic… you are not worried that anyone will see you* ” (Female Nurse IDI, clinic nurse).

It was felt that HCWs deserve special treatment because they are the ones who provide healthcare to others: *“… it is important because many HCW will get helped as a result they will be able to help others that are sick.”* (Female Nurse IDI, clinic patient). Close family members should also be beneficiaries for integrated family care: “ *… the closest relatives of staff members should also use the clinic because it is the staff who will be caring for the… if they [relatives] are not well cared for, the staff themselves may be absent from work at the hospital …we already have such a shortage of workers*” (Hospital Management, IDI). Finally, it was mentioned that most of the people working within the public health sector could not afford to go to private clinics where they could obtain more private care: *“… it is very important that people should know this clinic, because for people like us we cannot manage to go to the private clinics to receive treatment…”* (Female Clinical Officer FGD)

### Perceived Barriers to Uptake of Services at HCW HIV Clinic

Respondents perceived that there were still a substantial number of HCW who had not yet utilized the confidential HIV testing service. Some respondents indicated that HCW should also be encouraged to go for HTC with the knowledge that confidential services are available: *“…sometimes we people…know our status but fail to go there to get the help that we need because we are afraid that other people would see us. So when we come to come to the hospital, it is at a time when we are very sick and there is really nothing much that our colleagues can do to us anymore.”* (Female Nurse IDI, clinic nurse)

Privacy and confidentiality were the major concerns of interviewees noted as hampering the uptake of services at the clinic. Despite the fact that so many steps were taken to address confidentiality, respondents feared of being seen by their fellow colleagues who are not HIV positive. Some respondents highlighted that clients were fearful of being tested and were in denial about accepting their HIV test results. Distance from the rural health centers and lack of transport to and from the HCW HIV clinic was mentioned by a few participants, some respondents perceived no barriers to the use of the clinic.

Respondents were asked to suggest ways of addressing the concerns or barriers raised in the former section and suggested: sensitization meetings to address the issue of lack of awareness; further isolating the clinic; maintaining confidentiality; increasing the numbers of rooms and number of clinic staff; assure users about the sustainability of the HCW HIV clinic. Some respondents had strong views that the clinic needed to be isolated to a more isolated place elsewhere. One of the attending clients suggested the exclusion of HCW that are not HIV infected from providing services to HIV infected staff. A suggestion was also made to have a general integrated HCW HIV clinic where HIV related services were integrated and not separate.

## Discussion

Program retention (number alive and on treatment) and rates of defaulting off treatment at the HCW HIV clinic appears to be significantly better than at the general HIV clinic. In univariate analysis, mortality was significantly lower in the HCW HIV clinic however we were unable to demonstrate an association in multivariate analysis. Under-reporting of mortality in the general HIV clinic may occur due to misclassifcation of mortality as lost-to-follow up. These findings may be explained by differences in quality of care provided in each setting. Quality of care may be better in the HCW HIV clinic due to lower patient volume and individual caregiver workload (15–20 patient visits per provider per day compared with 60–75 patient visits per provider per day in the general clinic [17]). Other contributing factors to quality include the presence and consistency of one nurse who is familiar with all of the patients, and the availability of a senior consultant clinician. In addition, the patient cohort itself could provides a possible selection bias since HCW's higher baseline knowledge of HIV could lead to earlier recognition of concerning symptoms which may be associated with both decreased morbidity and mortality. A previous paper [15] describing the general cohort demonstrated a five fold increase in defaulting in those who experienced treatment related side effects after 18 months of follow up (almost certainly due to the use of a d4T in this setting). The difference in early side effect detection in the HCW HIV clinic, will also contribute to some degree to the differences in defaulting between the HCW HIV clinic and the general clinic. Proximity to the care site and access to transport also likely plays a role in these findings.

Secondary outcomes of side effects and substitution are also higher than the general population and significantly higher than national program statistics from the Malawi Ministry of Health, which at December 2008 had a side effect reporting rate of 4% and a substitution rate of 4% [18]. Of note, there was no difference in documentation of side effect based on the reported occupation of the HCW and whether or not the occupation would make them more likely to be familiar with HIV related complaints (i.e. support staff vs. directly involved in patient care). The implication is that there may be under-reporting or documentation of d4T side effects in situations where resource and time limitation may limit the ability of clinicians to detect and document d4T associated toxicity and substitute patients to less toxic medications. The rates of switching due to treatment failure are higher in the HCW HIV clinic due to increased detection because of patient and staff symptom recognition and also due to prioritization of immunological monitoring, with CD4s ordered at 6 monthly or 1-year intervals (pending availability) for HCW patients, which was not a part of the simplified approach adopted by the MOH due to lack of lab capacity. Finally, more people are initiated on ART on the basis of CD4 as opposed to WHO Clinical Stage 3/4, which may be related to earlier detection of indications for initiation of ART due to prioritization of and improved access to lab monitoring for HCW.

A previous qualitative study done in Uganda looking at reasons for delayed access to treatment amongst HIV positive HCW included: fear of real or perceived stigma, lack of prioritized HIV services, long clinic waiting times, fear of breach of confidentiality, lack of supportive social/legal environment, inadequate HIV/AIDS policies, lack of materials to augment universal precautions and protection against occupational exposure, and lack of prioritized eligibility criteria for ART [19]. Our qualitative study found that introduction of an HCW targeted HIV clinic led to awareness of clinic services and also corroborated that many HCW may not be accessing services due to issues of denial and acceptance, stigma and confidentiality. HCW perceived that the HCW HIV clinic was important in terms of prompt and high quality confidential care, reduced waiting times and convenience leading to less absenteeism (from being sick and also from missing work due to appointments and long general HIV clinic waits).

The utility of having a HCW targeted HIV clinic and it's impact on absenteeism and caregiver health has been noted by hospital management with the establishment of a concurrent HCW general outpatient clinic, the institution of Hepatitis B staff vaccination days at the HCW HIV clinic, and the institution of annual staff TB screening days at the HCW clinic. In countries like Malawi, the impact of HIV on the health workforce is clearly multi-factorial and ranges from workload related exhaustion and caregiver burnout, to morbidity and mortality from infection. HCW that are seropositive may not want their status to be known due to stigma and denial, and are thought to access health services later in disease progression [20]. Provision of care and treatment to HIV infected HCWs is a priority and a starting point for provision of ARV in resource constrained settings.

The major strength of the study is that the data is from operational program information collected as part of routine monitoring and evaluation from a national public system. However, study limitations include the retrospective nature of the data and concerns regarding accuracy and consistency that are associated with use of operational data. In addition, because data was extracted from routine monitoring and evaluation indicators, information that might affect outcomes (e.g. viral load, CD4, education level and markers of socio-economic status) is not available, as it is not a part of routine clinical care for ART provision under the Malawi MOH public health model. Further investigation and cost effectiveness of HCW targeted interventions is needed, in the future, to assess the impact (including cost) on the human resources for health crisis in high HIV prevalence, low resource settings.
